# Supplementary material for: Glacial allopatry vs. postglacial parapatry and peripatry: the case of hedgehogs
Source: PeerJ. 2017 Apr 25;5:e3163. doi: 10.7717/peerj.3163 (PMC5407276; doi:10.7717/peerj.3163)
Supplement: Table S1 — Given are: country of origin, haplotype code, colour code in Geneland analysis, sucess of microsatellite gonotyping, used code for individual, coordinates and collectors. [file peerj-05-3163-s001.docx]

| **Country of origin** | **Haplotype** | **Geneland** | **STR** |  | **N** | **E** | **Lgt** |  |  |  |  |  |  |  |  |  |  |  |  |  |  |  |  |  |  |
| --- | --- | --- | --- | --- | --- | --- | --- | --- | --- | --- | --- | --- | --- | --- | --- | --- | --- | --- | --- | --- | --- | --- | --- | --- | --- |
| Bosnia and Herzegovina | Hap_5 | green | no | BH12 | 43.18 | 18.49 | Kryštufek Boris | |  |  |  |  |  |  |  |  |  |  |  |  |  |  |  |  |  |
| Bulgaria | Hap_14 | green | no | BLG2 | 42.57 | 22.70 | Dundarová Heliana | |  |  |  |  |  |  |  |  |  |  |  |  |  |  |  |  |  |
| Croatia | Hap_41 | green | no | CR10 | 43.53 | 16.63 | Koren Toni | |  |  |  |  |  |  |  |  |  |  |  |  |  |  |  |  |  |
| Greece | Hap_17 | green | no | GR3 | 39.43 | 19.94 | Šandera Martin | |  |  |  |  |  |  |  |  |  |  |  |  |  |  |  |  |  |
| Greece | Hap_15 | green | no | GR4 | 39.47 | 19.90 | Šandera Martin | |  |  |  |  |  |  |  |  |  |  |  |  |  |  |  |  |  |
| Greece | Hap_1 | blue | no | GR18 | 41.09 | 22.32 | Šťáhlavský František, Hynková Ivana | | | |  |  |  |  |  |  |  |  |  |  |  |  |  |  |  |
| Greece | Hap_34 | blue | no | GR72 | 38.15 | 21.62 | NHMC Crete | |  |  |  |  |  |  |  |  |  |  |  |  |  |  |  |  |  |
| Greece | Hap_47 | blue | no | GR75 | 39.00 | 23.20 | NHMC Crete | |  |  |  |  |  |  |  |  |  |  |  |  |  |  |  |  |  |
| Greece | Hap_23 | blue | no | GR85 | 41.13 | 26.23 | NHMC Crete | |  |  |  |  |  |  |  |  |  |  |  |  |  |  |  |  |  |
| Greece | Hap_31 | blue | no | GR91 | 38.91 | 24.57 | NHMC Crete | |  |  |  |  |  |  |  |  |  |  |  |  |  |  |  |  |  |
| Greece | Hap_33 | blue | no | GR95 | 37.16 | 22.43 | NHMC Crete | |  |  |  |  |  |  |  |  |  |  |  |  |  |  |  |  |  |
| Greece | Hap_30 | blue | no | GR92 | 38.78 | 24.55 | NHMC Crete | |  |  |  |  |  |  |  |  |  |  |  |  |  |  |  |  |  |
| Crete | Hap_30 | blue | no | GR36 | 35.27 | 24.48 | NHMC Crete | |  |  |  |  |  |  |  |  |  |  |  |  |  |  |  |  |  |
| Crete | Hap_30 | blue | no | GR39 | 35.19 | 25.28 | NHMC Crete | |  |  |  |  |  |  |  |  |  |  |  |  |  |  |  |  |  |
| Crete | Hap_32 | blue | no | GR40 | 35.12 | 25.50 | NHMC Crete | |  |  |  |  |  |  |  |  |  |  |  |  |  |  |  |  |  |
| Crete | Hap_30 | blue | no | GR41 | 35.42 | 25.2 | NHMC Crete | |  |  |  |  |  |  |  |  |  |  |  |  |  |  |  |  |  |
| Crete | Hap_30 | blue | no | GR44 | 35.24 | 24.82 | NHMC Crete | |  |  |  |  |  |  |  |  |  |  |  |  |  |  |  |  |  |
| Crete | Hap_30 | blue | no | GR45 | 35.29 | 25.14 | NHMC Crete | |  |  |  |  |  |  |  |  |  |  |  |  |  |  |  |  |  |
| Crete | Hap_30 | blue | no | GR49 | 35.28 | 25.19 | NHMC Crete | |  |  |  |  |  |  |  |  |  |  |  |  |  |  |  |  |  |
| Crete | Hap_30 | blue | no | GR53 | 35.33 | 25.26 | NHMC Crete | |  |  |  |  |  |  |  |  |  |  |  |  |  |  |  |  |  |
| Crete | Hap_44 | blue | no | GR55 | 35.34 | 25.8 | NHMC Crete | |  |  |  |  |  |  |  |  |  |  |  |  |  |  |  |  |  |
| Crete | Hap_30 | blue | no | GR57 | 35.56 | 24.6 | NHMC Crete | |  |  |  |  |  |  |  |  |  |  |  |  |  |  |  |  |  |
| Crete | Hap_30 | blue | no | GR59 | 35.12 | 25.88 | NHMC Crete | |  |  |  |  |  |  |  |  |  |  |  |  |  |  |  |  |  |
| Crete | Hap_30 | blue | no | GR61 | 35.45 | 24.17 | NHMC Crete | |  |  |  |  |  |  |  |  |  |  |  |  |  |  |  |  |  |
| Crete | Hap_30 | blue | no | GR63 | 35.58 | 24.14 | NHMC Crete | |  |  |  |  |  |  |  |  |  |  |  |  |  |  |  |  |  |
| Crete | Hap_30 | blue | no | GR64 | 35.30 | 25.15 | NHMC Crete | |  |  |  |  |  |  |  |  |  |  |  |  |  |  |  |  |  |
| Crete | Hap_30 | blue | no | GR65 | 35.33 | 25.26 | NHMC Crete | |  |  |  |  |  |  |  |  |  |  |  |  |  |  |  |  |  |
| Crete | Hap_30 | blue | no | GR70 | 35.19 | 25.25 | NHMC Crete | |  |  |  |  |  |  |  |  |  |  |  |  |  |  |  |  |  |
| Crete | Hap_30 | blue | no | GR73 | 35.30 | 25.52 | NHMC Crete | |  |  |  |  |  |  |  |  |  |  |  |  |  |  |  |  |  |
| Italy | Hap_52 | pink | no | IT2 | 46.37 | 11.36 | Konečný |  |  |  |  |  |  |  |  |  |  |  |  |  |  |  |  |  |  |
| Italy | Hap_52 | pink | no | IT3 | 46.21 | 11.12 | Konečný |  |  |  |  |  |  |  |  |  |  |  |  |  |  |  |  |  |  |
| Macedony | Hap_17 | green | no | MC2 | 41.04 | 21.34 | Kryštufek Boris | |  |  |  |  |  |  |  |  |  |  |  |  |  |  |  |  |  |
| Macedony | Hap_2 | green | no | MC3 | 41.04 | 21.34 | Kryštufek Boris | |  |  |  |  |  |  |  |  |  |  |  |  |  |  |  |  |  |
| Macedony | Hap_21 | green | no | MC13 | 41.38 | 22.21 | Kryštufek Boris | |  |  |  |  |  |  |  |  |  |  |  |  |  |  |  |  |  |
| Macedony | Hap_19 | green | no | MC19 | 41.11 | 20.80 | Bolfíková Barbora | |  |  |  |  |  |  |  |  |  |  |  |  |  |  |  |  |  |
| Romania | Hap_23 | purple | no | RMN22 | 44.88 | 28.83 | Sándor D. Attila | |  |  |  |  |  |  |  |  |  |  |  |  |  |  |  |  |  |
| Romania | Hap_2 | yellow | no | RMN27 | 47.87 | 26.11 | Mihalca D. Andrei | |  |  |  |  |  |  |  |  |  |  |  |  |  |  |  |  |  |
| Romania | Hap_10 | yellow | no | RMN28 | 46.70 | 24.6 | Mihalca D. Andrei | |  |  |  |  |  |  |  |  |  |  |  |  |  |  |  |  |  |
| Romania | Hap_40 | yellow | no | RMN31 | 47.60 | 24.7 | Sándor D. Attila | |  |  |  |  |  |  |  |  |  |  |  |  |  |  |  |  |  |
| Romania | Hap_12 | yellow | no | RMN32 | 46.50 | 24.92 | Sándor D. Attila | |  |  |  |  |  |  |  |  |  |  |  |  |  |  |  |  |  |
| Romania | Hap_12 | yellow | no | RMN35 | 48.60 | 27.7 | Sándor D. Attila | |  |  |  |  |  |  |  |  |  |  |  |  |  |  |  |  |  |
| Romania | Hap_9 | yellow | no | RMN40 | 47.05 | 22.42 | Sándor D. Attila | |  |  |  |  |  |  |  |  |  |  |  |  |  |  |  |  |  |
| Romania | Hap_23 | purple | no | RMN43 | 44.55 | 28.20 | Mihalca D. Andrei | |  |  |  |  |  |  |  |  |  |  |  |  |  |  |  |  |  |
| Romania | Hap_10 | yellow | no | RMN44 | 46.73 | 23.36 | Sándor D. Attila | |  |  |  |  |  |  |  |  |  |  |  |  |  |  |  |  |  |
| Romania | Hap_8 | yellow | no | RMN46 | 46.019 | 23.54 | Mihalca D. Andrei | |  |  |  |  |  |  |  |  |  |  |  |  |  |  |  |  |  |
| Romania | Hap_4 | purple | no | RMN47 | 45.75 | 25.77 | Sándor Attila | |  |  |  |  |  |  |  |  |  |  |  |  |  |  |  |  |  |
| Romania | Hap_7 | purple | no | RMN5 | 44.10 | 28.3 | Sándor D. Attila | |  |  |  |  |  |  |  |  |  |  |  |  |  |  |  |  |  |
| Slovenia | Hap_17 | pink | no | SL9 | 46.19 | 14.40 | Kryštufek Boris | |  |  |  |  |  |  |  |  |  |  |  |  |  |  |  |  |  |
| Slovenia | Hap_17 | pink | no | SL14 | 45.51 | 13.59 | Kryštufek Boris | |  |  |  |  |  |  |  |  |  |  |  |  |  |  |  |  |  |
| Slovenia | Hap_17 | pink | no | SL18 | 46.11 | 14.14 | Kryštufek Boris | |  |  |  |  |  |  |  |  |  |  |  |  |  |  |  |  |  |
| Slovenia | Hap_32 | pink | no | SL21 | 46.59 | 16.1 | Črne Mitja | |  |  |  |  |  |  |  |  |  |  |  |  |  |  |  |  |  |
| Slovenia | Hap_17 | pink | no | SL34 | 45.55 | 13.73 | Zagoršek Tjaša | |  |  |  |  |  |  |  |  |  |  |  |  |  |  |  |  |  |
| Slovenia | Hap_42 | pink | no | SL6 | 45.87 | 14.82 | Kryštufek Boris | |  |  |  |  |  |  |  |  |  |  |  |  |  |  |  |  |  |
| Slovakia | Hap_1 | yellow | no | sk19 | 49.30 | 21.26 | Celuch Martin |  |  |  |  |  |  |  |  |  |  |  |  |  |  |  |  |  |  |
| Bosnia and Herzegovina | Hap_50 | green | yes | BH1 | 43.26 | 18.11 | Kryštufek Boris | 138 | 140 | 162 | 166 | 176 | 183 | 281 | 285 | 94 | 94 | 155 | 166 | 144 | 156 | 248 | 248 | 281 | 281 |
| Bosnia and Herzegovina | Hap_50 | green | yes | BH10 | 43.23 | 18.46 | Kryštufek Boris | 125 | 140 | 140 | 162 | 168 | 178 | 281 | 285 | 94 | 94 | 151 | 168 | 147 | 184 | 250 | 252 | 281 | 281 |
| Bosnia and Herzegovina | Hap_5 | pink | yes | BH11 | 44.55 | 16.37 | Kryštufek Boris | 129 | 129 | 159 | 164 | 178 | 189 | 285 | 285 | 94 | 94 | 157 | 168 | 149 | 149 | 239 | 246 | 272 | 276 |
| Bosnia and Herzegovina | Hap_42 | green | yes | BH13 | 43.72 | 17.22 | Kryštufek Boris | 136 | 136 | 162 | 164 | 176 | 183 | 281 | 281 | 94 | 94 | 159 | 168 | 147 | 160 | 248 | 248 | 276 | 281 |
| Bosnia and Herzegovina | Hap_5 | green | yes | BH14 | 43.83 | 17.1 | Kryštufek Boris | 125 | 136 | 164 | 168 | 180 | 187 | 281 | 285 | 94 | 94 | 0 | 0 | 149 | 149 | 0 | 0 | 272 | 276 |
| Bosnia and Herzegovina | Hap_42 | pink | yes | BH15 | 44.83 | 15.84 | Kryštufek Boris | 129 | 136 | 161 | 162 | 178 | 180 | 281 | 285 | 94 | 94 | 155 | 155 | 149 | 149 | 239 | 239 | 276 | 288 |
| Bosnia and Herzegovina | Hap_42 | green | yes | BH16 | 43.46 | 17.39 | Kryštufek Boris | 134 | 138 | 159 | 164 | 180 | 184 | 281 | 285 | 94 | 94 | 153 | 153 | 147 | 147 | 244 | 252 | 270 | 276 |
| Bosnia and Herzegovina | Hap_50 | green | yes | BH17 | 43.17 | 18.53 | Kryštufek Boris | 138 | 140 | 162 | 166 | 176 | 183 | 281 | 285 | 94 | 94 | 153 | 166 | 147 | 156 | 246 | 248 | 0 | 0 |
| Bosnia and Herzegovina | Hap_1 | green | yes | BH18 | 43.37 | 17.84 | Kryštufek Boris | 132 | 140 | 151 | 159 | 176 | 176 | 281 | 285 | 94 | 100 | 155 | 166 | 144 | 156 | 239 | 248 | 281 | 281 |
| Bosnia and Herzegovina | Hap_5 | green | yes | BH19 | 42.81 | 18.14 | Kryštufek Boris | 136 | 136 | 166 | 166 | 176 | 176 | 281 | 281 | 94 | 94 | 155 | 159 | 147 | 172 | 239 | 252 | 0 | 0 |
| Bosnia and Herzegovina | Hap_1 | green | yes | BH2 | 43.34 | 17.81 | Kryštufek Boris | 132 | 140 | 151 | 159 | 176 | 176 | 281 | 285 | 94 | 100 | 155 | 166 | 144 | 156 | 248 | 248 | 281 | 281 |
| Bosnia and Herzegovina | Hap_5 | green | yes | BH20 | 43.25 | 17.84 | Koren Toni | 129 | 129 | 159 | 168 | 176 | 184 | 281 | 287 | 94 | 94 | 159 | 168 | 144 | 172 | 239 | 248 | 276 | 283 |
| Bosnia and Herzegovina | Hap_8 | green | yes | BH21 | 43.16 | 18.54 | Kryštufek Boris | 125 | 127 | 162 | 162 | 176 | 180 | 281 | 281 | 94 | 94 | 166 | 168 | 147 | 151 | 239 | 248 | 0 | 0 |
| Bosnia and Herzegovina | Hap_5 | green | yes | BH22 | 43.26 | 18.12 | Kryštufek Boris | 129 | 140 | 151 | 164 | 189 | 189 | 285 | 287 | 94 | 94 | 155 | 159 | 147 | 147 | 239 | 248 | 0 | 0 |
| Bosnia and Herzegovina | Hap_22 | green | yes | BH23 | 43.63 | 17.5 | Kryštufek Boris | 129 | 140 | 164 | 166 | 174 | 178 | 0 | 0 | 94 | 94 | 155 | 174 | 147 | 147 | 246 | 250 | 0 | 0 |
| Bosnia and Herzegovina | Hap_2 | green | yes | BH24 | 43.26 | 18.12 | Gvoždík Václav | 129 | 136 | 140 | 151 | 176 | 176 | 285 | 285 | 94 | 94 | 159 | 166 | 147 | 153 | 244 | 248 | 272 | 281 |
| Bosnia and Herzegovina | Hap_17 | pink | yes | BH25 | 44.66 | 18.39 | Gvoždík Václav | 138 | 138 | 162 | 168 | 176 | 180 | 283 | 285 | 94 | 94 | 155 | 159 | 158 | 172 | 246 | 248 | 272 | 278 |
| Bosnia and Herzegovina | Hap_5 | green | yes | BH3 | 43.17 | 18.54 | Kryštufek Boris | 132 | 136 | 140 | 170 | 176 | 183 | 281 | 285 | 94 | 94 | 159 | 168 | 147 | 160 | 248 | 248 | 276 | 281 |
| Bosnia and Herzegovina | Hap_5 | green | yes | BH4 | 43.27 | 17.56 | Kryštufek Boris | 125 | 134 | 164 | 164 | 183 | 184 | 285 | 285 | 94 | 94 | 155 | 157 | 149 | 156 | 239 | 239 | 0 | 0 |
| Bosnia and Herzegovina | Hap_42 | pink | yes | BH5 | 44.61 | 16.25 | Kryštufek Boris | 129 | 136 | 166 | 168 | 178 | 189 | 281 | 281 | 94 | 94 | 153 | 155 | 149 | 149 | 239 | 256 | 272 | 278 |
| Bosnia and Herzegovina | Hap_42 | pink | yes | BH6 | 44.53 | 16.77 | Kryštufek Boris | 136 | 136 | 161 | 162 | 178 | 193 | 281 | 285 | 94 | 94 | 153 | 172 | 149 | 158 | 246 | 246 | 281 | 292 |
| Bosnia and Herzegovina | Hap_42 | pink | yes | BH7 | 44.42 | 17.8 | Kryštufek Boris | 125 | 139 | 162 | 164 | 178 | 178 | 285 | 287 | 94 | 94 | 153 | 159 | 144 | 147 | 248 | 250 | 278 | 279 |
| Bosnia and Herzegovina | Hap_5 | green | yes | BH8 | 43.88 | 18.40 | Kryštufek Boris | 129 | 129 | 159 | 168 | 174 | 178 | 281 | 281 | 94 | 94 | 153 | 155 | 151 | 172 | 246 | 246 | 272 | 274 |
| Bosnia and Herzegovina | Hap_5 | green | yes | BH9 | 43.41 | 18.51 | Kryštufek Boris | 129 | 138 | 151 | 168 | 178 | 184 | 281 | 287 | 94 | 94 | 155 | 161 | 147 | 158 | 248 | 248 | 278 | 279 |
| Bulgaria | Hap_24 | purple | yes | BLG1 | 42.80 | 24.63 | Kryštufek Boris | 136 | 140 | 151 | 162 | 174 | 184 | 281 | 287 | 94 | 100 | 151 | 157 | 147 | 149 | 239 | 239 | 278 | 281 |
| Bulgaria | Hap_49 | purple | yes | BLG3 | 43.23 | 24.70 | Kryštufek Boris | 125 | 129 | 162 | 170 | 174 | 178 | 281 | 289 | 94 | 94 | 151 | 157 | 149 | 151 | 239 | 248 | 272 | 272 |
| Bulgaria | Hap_23 | purple | yes | BLG4 | 42.26 | 25.32 | Kryštufek Boris | 138 | 138 | 161 | 166 | 168 | 189 | 269 | 276 | 94 | 94 | 153 | 155 | 149 | 168 | 246 | 248 | 0 | 0 |
| Croatia | Hap_1 | green | yes | CR1 | 43.63 | 16.70 | Kryštufek Boris | 125 | 132 | 159 | 161 | 180 | 180 | 285 | 287 | 94 | 94 | 155 | 157 | 147 | 147 | 246 | 250 | 278 | 279 |
| Croatia | Hap_6 | pink | yes | CR11 | 45.60 | 16.4 | Koren Toni | 136 | 140 | 151 | 162 | 180 | 180 | 281 | 285 | 94 | 94 | 153 | 155 | 149 | 149 | 248 | 248 | 279 | 279 |
| Croatia | Hap_17 | pink | yes | CR13 | 45.45 | 13.64 | Koren Toni | 125 | 132 | 168 | 170 | 180 | 180 | 281 | 287 | 94 | 94 | 172 | 174 | 144 | 147 | 246 | 248 | 274 | 274 |
| Croatia | Hap_17 | pink | yes | CR14 | 45.84 | 18.79 | Koren Toni | 134 | 134 | 161 | 162 | 187 | 195 | 281 | 290 | 94 | 94 | 155 | 161 | 149 | 178 | 239 | 248 | 276 | 285 |
| Croatia | Hap_23 | green | yes | CR15 | 42.74 | 17.54 | Kryštufek Boris | 125 | 125 | 151 | 170 | 176 | 184 | 287 | 287 | 94 | 94 | 155 | 159 | 153 | 153 | 252 | 252 | 283 | 283 |
| Croatia | Hap_23 | green | yes | CR16 | 42.74 | 17.54 | Kryštufek Boris | 125 | 125 | 151 | 170 | 176 | 184 | 287 | 287 | 94 | 94 | 155 | 159 | 153 | 153 | 252 | 252 | 283 | 283 |
| Croatia |  |  | yes | CR17 | 42.74 | 17.54 | Kryštufek Boris | 136 | 136 | 159 | 159 | 183 | 187 | 281 | 285 | 94 | 94 | 153 | 163 | 149 | 170 | 248 | 248 | 272 | 279 |
| Croatia | Hap_41 | green | yes | CR18 | 42.67 | 18.8 | Kryštufek Boris | 132 | 136 | 162 | 164 | 176 | 178 | 0 | 0 | 94 | 94 | 155 | 155 | 147 | 160 | 246 | 254 | 0 | 0 |
| Croatia | Hap_17 | pink | yes | CR19 | 45.29 | 18.81 | Koren Toni | 129 | 132 | 153 | 170 | 184 | 184 | 281 | 281 | 94 | 94 | 151 | 155 | 147 | 147 | 248 | 248 | 279 | 279 |
| Croatia | Hap_1 | green | yes | CR2 | 43.63 | 16.70 | Kryštufek Boris | 132 | 134 | 159 | 164 | 178 | 180 | 285 | 285 | 94 | 94 | 153 | 157 | 149 | 172 | 239 | 248 | 272 | 288 |
| Croatia | Hap_50 | green | yes | CR20 | 42.52 | 18.37 | Kryštufek Boris | 127 | 132 | 151 | 151 | 180 | 184 | 281 | 287 | 94 | 94 | 155 | 159 | 153 | 153 | 246 | 248 | 274 | 274 |
| Croatia | Hap_5 | green | yes | CR21 | 42.52 | 18.37 | Kryštufek Boris | 132 | 140 | 164 | 168 | 176 | 176 | 276 | 287 | 94 | 94 | 155 | 157 | 156 | 156 | 239 | 254 | 279 | 279 |
| Croatia | Hap_1 | green | yes | CR3 | 43.70 | 16.64 | Kryštufek Boris | 125 | 127 | 159 | 164 | 178 | 183 | 281 | 287 | 94 | 94 | 157 | 157 | 149 | 149 | 239 | 248 | 274 | 278 |
| Croatia | Hap_50 | green | yes | CR4 | 42.87 | 17.59 | Kryštufek Boris | 132 | 136 | 162 | 170 | 176 | 189 | 285 | 285 | 94 | 94 | 157 | 159 | 147 | 156 | 239 | 248 | 279 | 285 |
| Croatia | Hap_17 | pink | yes | CR5 | 45.93 | 13.74 | Kryštufek Boris | 132 | 138 | 161 | 164 | 180 | 180 | 281 | 281 | 94 | 94 | 153 | 159 | 184 | 187 | 239 | 246 | 285 | 285 |
| Croatia | Hap_17 | pink | yes | CR6 | 45.93 | 13.74 | Kryštufek Boris | 132 | 138 | 161 | 164 | 180 | 180 | 281 | 281 | 94 | 94 | 153 | 159 | 184 | 187 | 239 | 246 | 0 | 0 |
| Croatia |  |  | yes | CR7 | 44.44 | 15.6 | Kryštufek Boris | 119 | 138 | 166 | 166 | 178 | 178 | 0 | 0 | 94 | 100 | 155 | 159 | 149 | 172 | 246 | 248 | 276 | 276 |
| Croatia | Hap_23 | green | yes | CR8 | 43.18 | 17.65 | Koren Toni | 127 | 140 | 166 | 170 | 176 | 176 | 281 | 285 | 94 | 94 | 155 | 166 | 149 | 172 | 239 | 248 | 279 | 283 |
| Croatia | Hap_5 | green | yes | CR9 | 43.09 | 17.72 | Koren Toni | 127 | 140 | 166 | 170 | 176 | 176 | 281 | 285 | 94 | 94 | 155 | 166 | 149 | 172 | 239 | 248 | 279 | 283 |
| Greece | Hap_35 | blue | yes | GR19 | 40.83 | 21.77 | Šťáhlavský František, Hynková Ivana | 129 | 134 | 140 | 159 | 176 | 184 | 281 | 285 | 94 | 100 | 153 | 163 | 172 | 174 | 250 | 256 | 274 | 281 |
| Greece |  |  | yes | GR20 | 39.48 | 19.23 | Šťáhlavský František, Peprný Miroslav | 125 | 134 | 140 | 159 | 176 | 184 | 281 | 285 | 94 | 100 | 153 | 163 | 172 | 174 | 250 | 256 | 274 | 281 |
| Greece | Hap_30 | blue | yes | GR54 | 35.36 | 24.48 | Kryštufek Boris | 136 | 138 | 159 | 159 | 180 | 184 | 281 | 281 | 94 | 94 | 168 | 170 | 156 | 156 | 246 | 246 | 274 | 276 |
| Greece |  |  | yes | GR79 | 41.13 | 26.23 | Kryštufek Boris | 125 | 140 | 162 | 164 | 183 | 191 | 281 | 281 | 94 | 94 | 155 | 155 | 144 | 149 | 246 | 248 | 0 | 0 |
| Greece | Hap_48 | violet | yes | GR80 | 41.13 | 26.23 | Kryštufek Boris | 125 | 132 | 164 | 168 | 184 | 189 | 281 | 285 | 94 | 94 | 151 | 155 | 151 | 151 | 246 | 248 | 272 | 276 |
| Greece | Hap_53 | blue | yes | GR86 | 35.33 | 25.11 | Kryštufek Boris | 125 | 138 | 155 | 161 | 176 | 183 | 281 | 289 | 94 | 100 | 161 | 161 | 144 | 144 | 246 | 246 | 0 | 0 |
| Greece | Hap_30 | blue | yes | GR87 | 35.27 | 25.18 | Kryštufek Boris | 129 | 138 | 159 | 159 | 180 | 180 | 290 | 292 | 94 | 100 | 155 | 157 | 153 | 156 | 246 | 246 | 274 | 278 |
| Greece | Hap_26 | blue | yes | GR89 | 35.37 | 24.53 | Kryštufek Boris | 129 | 129 | 159 | 159 | 178 | 180 | 290 | 290 | 94 | 94 | 148 | 155 | 153 | 153 | 246 | 246 | 274 | 276 |
| Greece | Hap_30 | blue | yes | GR90 | 35.33 | 25.28 | Kryštufek Boris | 129 | 132 | 162 | 164 | 183 | 184 | 281 | 281 | 94 | 94 | 155 | 163 | 147 | 176 | 246 | 248 | 272 | 274 |
| Crete | Hap_30 | blue | yes | GR34 | 35.49 | 23.69 | NHMC Crete | 129 | 136 | 159 | 161 | 180 | 180 | 290 | 290 | 100 | 100 | 155 | 157 | 156 | 158 | 246 | 246 | 270 | 276 |
| Crete | Hap_30 | blue | yes | GR35 | 35.25 | 24.81 | NHMC Crete | 129 | 134 | 159 | 161 | 180 | 180 | 290 | 292 | 94 | 100 | 148 | 155 | 153 | 158 | 246 | 260 | 272 | 276 |
| Crete | Hap_30 | blue | yes | GR37 | 35.30 | 25.16 | NHMC Crete | 134 | 134 | 159 | 162 | 180 | 183 | 287 | 290 | 94 | 100 | 148 | 155 | 153 | 158 | 246 | 246 | 0 | 0 |
| Crete | Hap_37 | blue | yes | GR42 | 37.10 | 25.38 | NHMC Crete | 132 | 134 | 159 | 159 | 180 | 183 | 290 | 290 | 0 | 0 | 148 | 155 | 153 | 153 | 0 | 0 | 0 | 0 |
| Crete | Hap_45 | blue | yes | GR43 | 35.49 | 23.69 | NHMC Crete | 129 | 134 | 159 | 159 | 178 | 180 | 281 | 290 | 100 | 100 | 155 | 155 | 153 | 153 | 246 | 246 | 272 | 272 |
| Crete | Hap_30 | blue | yes | GR47 | 35.38 | 24.21 | NHMC Crete | 132 | 134 | 159 | 159 | 180 | 180 | 290 | 292 | 100 | 100 | 155 | 155 | 153 | 153 | 246 | 246 | 0 | 0 |
| Crete | Hap_30 | blue | yes | GR48 | 35.34 | 25.14 | NHMC Crete | 129 | 129 | 159 | 159 | 183 | 184 | 281 | 292 | 94 | 100 | 155 | 155 | 153 | 156 | 246 | 246 | 0 | 0 |
| Crete | Hap_30 | blue | yes | GR50 | 35.25 | 25.14 | NHMC Crete | 134 | 138 | 159 | 159 | 180 | 183 | 292 | 292 | 94 | 94 | 155 | 163 | 153 | 153 | 246 | 246 | 0 | 0 |
| Crete | Hap_30 | blue | yes | GR51 | 35.30 | 25.16 | NHMC Crete | 132 | 134 | 159 | 161 | 180 | 183 | 290 | 290 | 94 | 94 | 148 | 155 | 161 | 161 | 246 | 246 | 274 | 274 |
| Crete | Hap_30 | blue | yes | GR52 | 35.05 | 25.6 | NHMC Crete | 132 | 134 | 157 | 159 | 180 | 184 | 281 | 281 | 94 | 94 | 155 | 155 | 153 | 153 | 246 | 246 | 272 | 276 |
| Crete | Hap_30 | blue | yes | GR56 | 35.24 | 24.81 | NHMC Crete | 132 | 134 | 159 | 161 | 178 | 180 | 290 | 290 | 94 | 100 | 155 | 157 | 158 | 161 | 246 | 246 | 0 | 0 |
| Crete | Hap_30 | blue | yes | GR62 | 35.19 | 25.25 | NHMC Crete | 127 | 134 | 159 | 159 | 180 | 180 | 290 | 290 | 94 | 94 | 155 | 155 | 153 | 168 | 246 | 246 | 276 | 276 |
| Crete | Hap_30 | blue | yes | GR66 | 35.27 | 25.11 | NHMC Crete | 132 | 134 | 159 | 159 | 180 | 183 | 276 | 292 | 100 | 100 | 148 | 155 | 153 | 153 | 246 | 246 | 274 | 279 |
| Crete | Hap_30 | blue | yes | GR67 | 41.13 | 26.23 | NHMC Crete | 129 | 138 | 159 | 159 | 180 | 187 | 276 | 292 | 100 | 100 | 148 | 155 | 153 | 153 | 246 | 246 | 274 | 276 |
| Crete | Hap_30 | blue | yes | GR68 | 41.13 | 26.23 | NHMC Crete | 134 | 136 | 159 | 164 | 180 | 180 | 290 | 290 | 94 | 94 | 155 | 155 | 156 | 156 | 246 | 246 | 268 | 270 |
| Crete | Hap_30 | blue | yes | GR69 | 35.19 | 25.25 | NHMC Crete | 134 | 134 | 159 | 159 | 180 | 184 | 290 | 294 | 100 | 100 | 155 | 155 | 153 | 158 | 246 | 246 | 272 | 276 |
| Crete | Hap_30 | blue | yes | GR74 | 35.33 | 25.26 | NHMC Crete | 134 | 134 | 159 | 159 | 180 | 180 | 290 | 290 | 94 | 94 | 148 | 148 | 153 | 153 | 246 | 246 | 272 | 276 |
| Crete | Hap_30 | blue | yes | GR77 | 35.12 | 25.34 | NHMC Crete | 138 | 140 | 159 | 159 | 180 | 180 | 292 | 292 | 100 | 100 | 148 | 155 | 158 | 158 | 246 | 246 | 0 | 0 |
| Crete | Hap_30 | blue | yes | GR78 | 39.19 | 22.76 | NHMC Crete | 134 | 136 | 157 | 159 | 178 | 180 | 0 | 0 | 100 | 100 | 155 | 155 | 153 | 153 | 246 | 246 | 0 | 0 |
| Crete | Hap_30 | blue | yes | GR81 | 38.48 | 22.58 | NHMC Crete | 134 | 136 | 159 | 159 | 178 | 183 | 281 | 290 | 100 | 100 | 148 | 148 | 153 | 153 | 246 | 246 | 272 | 272 |
| Crete | Hap_30 | blue | yes | GR82 | 35.34 | 25.12 | NHMC Crete | 134 | 134 | 159 | 159 | 180 | 180 | 290 | 292 | 94 | 94 | 148 | 155 | 158 | 158 | 246 | 246 | 274 | 274 |
| Crete | Hap_30 | blue | yes | GR83 | 39.29 | 22.17 | NHMC Crete | 129 | 134 | 157 | 159 | 178 | 180 | 0 | 0 | 100 | 100 | 148 | 148 | 153 | 153 | 0 | 0 | 0 | 0 |
| Crete |  |  | yes | GR88 | 39.56 | 20.79 | NHMC Crete | 129 | 143 | 159 | 172 | 178 | 183 | 281 | 283 | 94 | 100 | 153 | 166 | 147 | 156 | 246 | 246 | 272 | 272 |
| Crete | Hap_30 | blue | yes | GR94 | 35.31 | 25.15 | NHMC Crete | 121 | 123 | 159 | 159 | 180 | 183 | 290 | 290 | 100 | 100 | 155 | 155 | 153 | 153 | 246 | 246 | 276 | 276 |
| Hungary | Hap_27 | orange | yes | M10 | 47.49 | 19.22 | Rigó Krisztina | 136 | 136 | 159 | 159 | 184 | 184 | 281 | 283 | 94 | 94 | 144 | 144 | 144 | 168 | 250 | 250 | 272 | 272 |
| Hungary | Hap_17 | pink | yes | M11 | 45.99 | 18.7 | Kryštufek Boris | 134 | 134 | 162 | 162 | 184 | 187 | 281 | 290 | 94 | 94 | 159 | 159 | 149 | 151 | 246 | 248 | 274 | 276 |
| Hungary | Hap_40 | yellow | yes | M12 | 47.16 | 20.25 | Kryštufek Boris | 129 | 138 | 159 | 161 | 176 | 178 | 281 | 283 | 94 | 94 | 159 | 161 | 153 | 153 | 248 | 250 | 246 | 287 |
| Hungary | Hap_17 | pink | yes | M4 | 47.31 | 19.22 | Schneiderová Irena | 129 | 145 | 159 | 162 | 184 | 184 | 289 | 290 | 94 | 94 | 159 | 159 | 153 | 168 | 246 | 248 | 0 | 0 |
| Hungary | Hap_1 | orange | yes | M5 | 47.63 | 18.90 | Rigó Krisztina | 129 | 134 | 159 | 159 | 180 | 187 | 283 | 283 | 94 | 96 | 155 | 163 | 168 | 170 | 246 | 246 | 272 | 276 |
| Hungary | Hap_27 | orange | yes | M6 | 47.49 | 19.22 | Rigó Krisztina | 113 | 132 | 162 | 164 | 183 | 184 | 281 | 281 | 94 | 94 | 153 | 159 | 166 | 168 | 246 | 246 | 276 | 276 |
| Hungary | Hap_27 | orange | yes | M7 | 47.53 | 19.5 | Rigó Krisztina | 113 | 134 | 161 | 161 | 162 | 183 | 281 | 292 | 94 | 94 | 159 | 163 | 153 | 170 | 248 | 250 | 276 | 283 |
| Hungary | Hap_1 | orange | yes | M8 | 47.53 | 19.5 | Rigó Krisztina | 134 | 134 | 162 | 166 | 184 | 184 | 281 | 292 | 94 | 96 | 144 | 159 | 168 | 168 | 246 | 250 | 274 | 274 |
| Hungary | Hap_1 | orange | yes | M9 | 47.63 | 18.90 | Rigó Krisztina | 129 | 129 | 159 | 166 | 178 | 183 | 281 | 285 | 94 | 94 | 157 | 159 | 168 | 170 | 246 | 248 | 276 | 278 |
| Macedony | Hap_23 | green | yes | MC1 | 41.13 | 20.82 | Kryštufek Boris | 129 | 129 | 159 | 164 | 184 | 184 | 281 | 281 | 96 | 96 | 124 | 168 | 168 | 168 | 241 | 241 | 274 | 274 |
| Macedony | Hap_36 | green | yes | MC10 | 41.18 | 20.68 | Kryštufek Boris | 129 | 129 | 159 | 168 | 174 | 178 | 281 | 281 | 94 | 94 | 153 | 155 | 151 | 172 | 246 | 246 | 272 | 274 |
| Macedony | Hap_36 | green | yes | MC11 | 41.26 | 21.51 | Kryštufek Boris | 138 | 140 | 155 | 164 | 178 | 187 | 276 | 283 | 94 | 100 | 153 | 155 | 144 | 149 | 246 | 248 | 276 | 278 |
| Macedony | Hap_19 | green | yes | MC12 | 41.36 | 22.29 | Kryštufek Boris | 127 | 132 | 159 | 159 | 180 | 184 | 285 | 287 | 94 | 94 | 151 | 159 | 147 | 176 | 248 | 248 | 276 | 276 |
| Macedony | Hap_23 | green | yes | MC14 | 41.13 | 20.81 | Kryštufek Boris | 129 | 129 | 159 | 164 | 184 | 184 | 281 | 281 | 94 | 94 | 153 | 161 | 149 | 170 | 246 | 246 | 270 | 272 |
| Macedony | Hap_38 | green | yes | MC16 | 41.36 | 20.81 | Šťáhlavský František, Hynková Ivana | 127 | 140 | 164 | 164 | 183 | 184 | 281 | 281 | 94 | 94 | 151 | 161 | 144 | 156 | 246 | 248 | 0 | 0 |
| Macedony | |  | yes | MC18 | 42.00 | 21.34 | Bolfíková Barbora | 125 | 136 | 161 | 164 | 180 | 189 | 285 | 285 | 88 | 94 | 155 | 155 | 147 | 147 | 246 | 248 | 0 | 0 |
| Macedony | Hap_5 | green | yes | MC4 | 41.04 | 21.33 | Kryštufek Boris | 138 | 143 | 159 | 164 | 176 | 187 | 281 | 285 | 94 | 94 | 157 | 159 | 149 | 161 | 246 | 256 | 274 | 274 |
| Macedony | Hap_19 | green | yes | MC5 | 41.75 | 22.20 | Kryštufek Boris | 132 | 134 | 168 | 168 | 183 | 184 | 281 | 283 | 94 | 94 | 168 | 168 | 147 | 172 | 248 | 248 | 270 | 274 |
| Macedony | Hap_20 | green | yes | MC6 | 41.04 | 21.00 | Kryštufek Boris | 123 | 127 | 162 | 164 | 183 | 184 | 281 | 283 | 94 | 94 | 155 | 159 | 149 | 156 | 239 | 248 | 272 | 276 |
| Macedony | Hap_17 | green | yes | MC7 | 41.04 | 21.33 | Kryštufek Boris | 129 | 138 | 161 | 166 | 180 | 187 | 281 | 283 | 94 | 100 | 155 | 157 | 0 | 0 | 246 | 248 | 272 | 274 |
| Macedony | Hap_2 | green | yes | MC8 | 41.41 | 21.44 | Kryštufek Boris | 117 | 138 | 159 | 159 | 184 | 187 | 283 | 287 | 94 | 100 | 153 | 155 | 144 | 174 | 246 | 256 | 278 | 281 |
| Macedony | Hap_5 | green | yes | MC9 | 41.41 | 21.44 | Kryštufek Boris | 138 | 143 | 159 | 164 | 176 | 187 | 281 | 285 | 94 | 100 | 157 | 159 | 149 | 161 | 246 | 256 | 274 | 274 |
| Monte Negro | Hap_5 | green | yes | MN1 | 42.24 | 19.9 | Kryštufek Boris | 132 | 138 | 140 | 159 | 176 | 189 | 285 | 287 | 94 | 94 | 157 | 159 | 151 | 151 | 246 | 250 | 0 | 0 |
| Monte Negro | Hap_23 | green | yes | MN2 | 42.44 | 19.28 | Kryštufek Boris | 129 | 138 | 159 | 159 | 176 | 180 | 281 | 281 | 94 | 94 | 155 | 168 | 144 | 170 | 246 | 250 | 276 | 279 |
| Monte Negro | Hap_23 | green | yes | MN3 | 42.96 | 19.9 | Kryštufek Boris | 129 | 132 | 151 | 159 | 183 | 187 | 276 | 285 | 94 | 94 | 151 | 168 | 144 | 147 | 0 | 0 | 0 | 0 |
| Monte Negro | Hap_23 | green | yes | MN4 | 42.96 | 19.10 | Kryštufek Boris | 129 | 132 | 151 | 159 | 183 | 187 | 276 | 285 | 94 | 100 | 151 | 168 | 144 | 147 | 246 | 248 | 270 | 278 |
| Monte Negro | Hap_5 | green | yes | MN5 | 42.24 | 19.9 | Kryštufek Boris | 132 | 136 | 162 | 170 | 176 | 189 | 285 | 285 | 94 | 94 | 157 | 159 | 147 | 156 | 239 | 248 | 279 | 285 |
| Monte Negro | Hap_1 | green | yes | MN6 | 42.43 | 18.81 | Kryštufek Boris | 132 | 140 | 159 | 159 | 189 | 191 | 281 | 285 | 94 | 94 | 157 | 157 | 147 | 147 | 239 | 239 | 274 | 274 |
| Romania |  |  | yes | RMN10 | 44.64 | 28.70 | Sándor D. Attila | 138 | 140 | 161 | 162 | 183 | 187 | 281 | 281 | 94 | 94 | 155 | 159 | 144 | 149 | 246 | 248 | 0 | 0 |
| Romania | Hap_23 | purple | yes | RMN11 | 44.22 | 28.39 | Sándor D. Attila | 127 | 136 | 159 | 161 | 176 | 180 | 281 | 285 | 94 | 94 | 144 | 155 | 149 | 151 | 239 | 246 | 272 | 279 |
| Romania | Hap_23 | purple | yes | RMN12 | 45.08 | 28.74 | Sándor D. Attila | 127 | 136 | 159 | 170 | 183 | 189 | 281 | 283 | 94 | 94 | 153 | 155 | 144 | 149 | 246 | 248 | 0 | 0 |
| Romania | Hap_25 | purple | yes | RMN13 | 45.24 | 28.18 | Sándor D. Attila | 136 | 138 | 153 | 161 | 176 | 183 | 285 | 285 | 94 | 94 | 159 | 174 | 149 | 168 | 248 | 248 | 267 | 268 |
| Romania | Hap_1 |  | yes | RMN14 | 45.25 | 28.14 | Kryštufek Boris | 136 | 138 | 159 | 161 | 187 | 189 | 285 | 285 | 94 | 94 | 153 | 157 | 144 | 166 | 246 | 248 | 272 | 272 |
| Romania | Hap_23 | purple | yes | RMN15 | 44.93 | 28.45 | Sándor D. Attila | 127 | 138 | 159 | 162 | 0 | 0 | 0 | 0 | 94 | 100 | 153 | 155 | 144 | 172 | 239 | 239 | 0 | 0 |
| Romania | Hap_23 | purple | yes | RMN16 | 44.72 | 28.68 | Sándor D. Attila | 117 | 127 | 161 | 161 | 176 | 178 | 276 | 281 | 94 | 94 | 153 | 155 | 149 | 158 | 0 | 0 | 0 | 0 |
| Romania | Hap_11 | purple | yes | RMN17 | 45.16 | 28.79 | Sándor D. Attila | 134 | 143 | 159 | 166 | 180 | 180 | 281 | 285 | 94 | 94 | 153 | 155 | 151 | 151 | 246 | 248 | 263 | 274 |
| Romania | Hap_10 | yellow | yes | RMN18 | 46.83 | 23.22 | Sándor D. Attila | 102 | 136 | 166 | 166 | 158 | 189 | 283 | 283 | 94 | 96 | 151 | 153 | 144 | 144 | 246 | 246 | 279 | 279 |
| Romania | Hap_23 | purple | yes | RMN19 | 45.16 | 28.80 | Sándor D. Attila | 123 | 134 | 159 | 162 | 176 | 189 | 281 | 283 | 94 | 94 | 153 | 153 | 151 | 151 | 248 | 248 | 0 | 0 |
| Romania | Hap_13 | purple | yes | RMN20 | 45.52 | 26.23 | Sándor D. Attila | 136 | 140 | 168 | 172 | 0 | 0 | 0 | 0 | 94 | 94 | 144 | 155 | 170 | 170 | 250 | 250 | 270 | 278 |
| Romania | Hap_4 | purple | yes | RMN21 | 44.64 | 28.72 | Sándor D. Attila | 136 | 140 | 162 | 174 | 183 | 183 | 283 | 283 | 94 | 94 | 157 | 161 | 149 | 160 | 250 | 250 | 272 | 276 |
| Romania | Hap_4 | purple | yes | RMN23 | 44.88 | 28.83 | Sándor D. Attila | 101 | 136 | 159 | 166 | 183 | 183 | 0 | 0 | 94 | 94 | 157 | 157 | 144 | 147 | 248 | 250 | 272 | 272 |
| Romania | Hap_40 | yellow | yes | RMN24 | 46.11 | 25.5 | Sándor D. Attila | 138 | 140 | 159 | 161 | 187 | 189 | 281 | 285 | 94 | 94 | 151 | 151 | 144 | 144 | 248 | 248 | 276 | 278 |
| Romania | Hap_1 | yellow | yes | RMN25 | 46.46 | 24.3 | Sándor D. Attila | 132 | 136 | 159 | 162 | 164 | 184 | 0 | 0 | 96 | 96 | 153 | 161 | 147 | 166 | 246 | 246 | 272 | 272 |
| Romania | Hap_25 | purple | yes | RMN26 | 44.88 | 28.75 | Sándor D. Attila | 136 | 136 | 162 | 170 | 183 | 183 | 0 | 0 | 94 | 94 | 155 | 159 | 149 | 149 | 0 | 0 | 0 | 0 |
| Romania | Hap_9 | yellow | yes | RMN29 | 47.82 | 24.23 | Sándor D. Attila | 125 | 127 | 159 | 159 | 187 | 187 | 281 | 281 | 94 | 94 | 157 | 161 | 144 | 144 | 248 | 248 | 0 | 0 |
| Romania | Hap_40 | yellow | yes | RMN33 | 46.63 | 25.5 | Sándor D. Attila | 136 | 143 | 162 | 164 | 178 | 184 | 283 | 283 | 94 | 94 | 157 | 166 | 147 | 147 | 248 | 248 | 0 | 0 |
| Romania | Hap_40 | yellow | yes | RMN34 | 46.52 | 25.15 | Sándor D. Attila | 127 | 134 | 155 | 172 | 178 | 183 | 287 | 287 | 96 | 100 | 155 | 161 | 144 | 166 | 246 | 252 | 0 | 0 |
| Romania | Hap_40 | yellow | yes | RMN36 | 46.60 | 25.7 | Sándor D. Attila | 117 | 136 | 164 | 166 | 178 | 178 | 0 | 0 | 94 | 94 | 151 | 155 | 144 | 144 | 0 | 0 | 0 | 0 |
| Romania | Hap_40 | yellow | yes | RMN37 | 46.45 | 24.30 | Kryštufek Boris | 134 | 136 | 162 | 164 | 184 | 187 | 281 | 285 | 94 | 96 | 161 | 161 | 144 | 144 | 248 | 250 | 0 | 0 |
| Romania | Hap_9 | yellow | yes | RMN38 | 46.54 | 23.78 | Sándor D. Attila | 132 | 138 | 159 | 161 | 191 | 193 | 0 | 0 | 0 | 0 | 161 | 161 | 147 | 153 | 250 | 250 | 0 | 0 |
| Romania | Hap_51 | yellow | yes | RMN39 | 47.05 | 22.42 | Sándor D. Attila | 119 | 134 | 159 | 161 | 183 | 183 | 281 | 281 | 94 | 96 | 148 | 157 | 147 | 147 | 246 | 250 | 270 | 270 |
| Romania | Hap_4 | purple | yes | RMN4 | 45.14 | 27.57 | Sándor D. Attila | 132 | 138 | 166 | 170 | 180 | 180 | 0 | 0 | 94 | 94 | 157 | 166 | 144 | 149 | 248 | 248 | 0 | 0 |
| Romania | Hap_8 | yellow | yes | RMN41 | 46.53 | 24.96 | Sándor D. Attila | 134 | 136 | 159 | 164 | 184 | 184 | 281 | 287 | 96 | 96 | 161 | 161 | 144 | 144 | 248 | 252 | 272 | 278 |
| Romania | Hap_1 | yellow | yes | RMN42 | 46.83 | 24.74 | Sándor D. Attila | 125 | 127 | 162 | 162 | 176 | 180 | 281 | 281 | 94 | 94 | 155 | 159 | 144 | 166 | 0 | 0 | 0 | 0 |
| Romania | Hap_23 | purple | yes | RMN45 | 45.26 | 28.9 | Sándor D. Attila | 127 | 134 | 162 | 170 | 176 | 191 | 281 | 283 | 94 | 94 | 151 | 155 | 149 | 151 | 246 | 248 | 0 | 0 |
| Romania | Hap_1 | yellow | yes | RMN48 | 46.96 | 22.72 | Ghira Ioan | 129 | 132 | 159 | 164 | 180 | 184 | 285 | 287 | 94 | 94 | 157 | 157 | 147 | 153 | 246 | 246 | 0 | 0 |
| Romania | Hap_4 | purple | yes | RMN6 | 45.12 | 26.88 | Sándor D. Attila | 119 | 119 | 166 | 166 | 178 | 193 | 285 | 287 | 94 | 94 | 157 | 157 | 144 | 160 | 248 | 248 | 268 | 270 |
| Romania | Hap_8 | yellow | yes | RMN8 | 46.47 | 24.15 | Sándor D. Attila | 132 | 136 | 159 | 164 | 174 | 183 | 0 | 0 | 96 | 96 | 155 | 155 | 147 | 166 | 250 | 250 | 272 | 272 |
| Romania | Hap_40 | yellow | yes | RMN9 | 46.77 | 24.70 | Sándor D. Attila | 127 | 134 | 161 | 162 | 162 | 193 | 285 | 285 | 94 | 94 | 157 | 157 | 144 | 166 | 246 | 250 | 0 | 0 |
| Slovenia | Hap_17 | pink | yes | SL1 | 47.82 | 18.35 | Kryštufek Boris | 129 | 143 | 170 | 170 | 178 | 184 | 281 | 287 | 94 | 94 | 153 | 161 | 147 | 170 | 239 | 246 | 270 | 276 |
| Slovenia | Hap_42 | pink | yes | SL10 | 48.18 | 17.6 | Kryštufek Boris | 136 | 136 | 170 | 170 | 180 | 180 | 281 | 281 | 94 | 94 | 151 | 161 | 147 | 156 | 246 | 256 | 276 | 276 |
| Slovenia | Hap_17 | pink | yes | SL11 | 48.95 | 20.56 | Kryštufek Boris | 129 | 136 | 159 | 170 | 176 | 183 | 281 | 281 | 94 | 94 | 153 | 153 | 149 | 156 | 244 | 246 | 272 | 276 |
| Slovenia | Hap_17 | pink | yes | SL12 | 48.95 | 20.56 | Kryštufek Boris | 129 | 136 | 159 | 170 | 176 | 183 | 281 | 281 | 94 | 94 | 153 | 153 | 149 | 156 | 244 | 246 | 272 | 276 |
| Slovenia | Hap_17 | pink | yes | SL13 | 48.95 | 20.56 | Kryštufek Boris | 136 | 136 | 159 | 166 | 180 | 184 | 281 | 281 | 94 | 94 | 153 | 157 | 147 | 149 | 246 | 256 | 276 | 288 |
| Slovenia | Hap_42 | pink | yes | SL15 | 48.95 | 20.56 | Kryštufek Boris | 125 | 129 | 159 | 174 | 180 | 180 | 281 | 281 | 94 | 94 | 151 | 153 | 144 | 149 | 248 | 248 | 276 | 276 |
| Slovenia | Hap_16 | pink | yes | SL16 | 45.52 | 13.75 | Kryštufek Boris | 129 | 132 | 159 | 172 | 180 | 180 | 281 | 281 | 94 | 94 | 155 | 157 | 149 | 172 | 248 | 256 | 272 | 272 |
| Slovenia | Hap_42 | pink | yes | SL17 | 45.90 | 14.80 | Kryštufek Boris | 132 | 143 | 159 | 166 | 178 | 184 | 281 | 281 | 94 | 94 | 153 | 153 | 144 | 153 | 250 | 250 | 272 | 283 |
| Slovenia | Hap_17 | pink | yes | SL19 | 46.07 | 14.61 | Koren Toni | 125 | 125 | 164 | 170 | 178 | 178 | 281 | 292 | 94 | 94 | 155 | 159 | 147 | 172 | 0 | 0 | 272 | 281 |
| Slovenia | Hap_27 | pink | yes | SL20 | 45.79 | 14.99 | Kryštufek Boris | 129 | 132 | 162 | 166 | 178 | 180 | 281 | 281 | 94 | 94 | 155 | 157 | 149 | 153 | 239 | 248 | 0 | 0 |
| Slovenia | Hap_23 | pink | yes | SL22 | 45.75 | 15.55 | Kryštufek Boris | 125 | 125 | 170 | 170 | 176 | 176 | 285 | 285 | 94 | 94 | 155 | 159 | 153 | 168 | 252 | 252 | 279 | 279 |
| Slovenia | Hap_17 | pink | yes | SL23 | 45.87 | 14.82 | Kryštufek Boris | 129 | 132 | 162 | 170 | 180 | 183 | 281 | 285 | 94 | 94 | 155 | 157 | 149 | 172 | 239 | 256 | 276 | 283 |
| Slovenia | Hap_43 | pink | yes | SL24 | 46.09 | 14.9 | Kryštufek Boris | 132 | 136 | 164 | 172 | 180 | 183 | 281 | 281 | 94 | 94 | 153 | 157 | 147 | 156 | 246 | 248 | 0 | 0 |
| Slovenia | Hap_17 | pink | yes | SL25 | 46.71 | 16.20 | Kryštufek Boris | 129 | 136 | 159 | 159 | 180 | 180 | 281 | 281 | 94 | 94 | 153 | 159 | 149 | 156 | 246 | 248 | 276 | 276 |
| Slovenia | Hap_43 | pink | yes | SL26 | 42.81 | 18.14 | Kryštufek Boris | 125 | 136 | 162 | 162 | 180 | 187 | 285 | 285 | 94 | 94 | 153 | 161 | 147 | 172 | 246 | 256 | 279 | 285 |
| Slovenia | Hap_42 | pink | yes | SL27 | 46.66 | 15.93 | Kryštufek Boris | 129 | 129 | 159 | 166 | 180 | 184 | 281 | 281 | 94 | 94 | 148 | 157 | 149 | 149 | 248 | 248 | 276 | 279 |
| Slovenia | Hap_42 | pink | yes | SL28 | 45.81 | 14.97 | Kryštufek Boris | 129 | 136 | 159 | 164 | 184 | 187 | 281 | 299 | 94 | 94 | 157 | 157 | 147 | 170 | 248 | 256 | 276 | 278 |
| Slovenia | Hap_42 | pink | yes | SL29 | 45.78 | 15.28 | Kryštufek Boris | 125 | 136 | 159 | 164 | 180 | 184 | 281 | 281 | 94 | 100 | 153 | 155 | 156 | 170 | 239 | 244 | 0 | 0 |
| Slovenia | Hap_17 | pink | yes | SL3 | 45.61 | 15.25 | Kryštufek Boris | 134 | 134 | 159 | 159 | 158 | 176 | 283 | 287 | 94 | 94 | 157 | 157 | 147 | 176 | 250 | 250 | 272 | 279 |
| Slovenia | Hap_17 | pink | yes | SL30 | 46.16 | 14.42 | Kryštufek Boris | 129 | 136 | 162 | 162 | 172 | 183 | 281 | 281 | 94 | 94 | 151 | 159 | 147 | 149 | 248 | 256 | 274 | 281 |
| Slovenia | Hap_42 | pink | yes | SL31 | 45.89 | 15.57 | Kryštufek Boris | 132 | 136 | 159 | 159 | 172 | 183 | 281 | 287 | 94 | 94 | 153 | 157 | 156 | 170 | 244 | 248 | 274 | 276 |
| Slovenia | Hap_42 | pink | yes | SL32 | 46.52 | 15.59 | Kryštufek Boris | 134 | 134 | 170 | 172 | 172 | 180 | 281 | 285 | 94 | 100 | 153 | 157 | 144 | 147 | 246 | 256 | 278 | 283 |
| Slovenia | Hap_17 | pink | yes | SL33 | 46.51 | 15.8 | Zagoršek Tjaša | 132 | 136 | 159 | 170 | 184 | 184 | 281 | 281 | 94 | 94 | 153 | 159 | 147 | 170 | 246 | 256 | 278 | 278 |
| Slovenia | Hap_17 | pink | yes | SL35 | 46.34 | 15.17 | Zagoršek Tjaša | 138 | 138 | 170 | 172 | 0 | 0 | 0 | 0 | 94 | 94 | 153 | 172 | 149 | 153 | 0 | 0 | 0 | 0 |
| Slovenia | Hap_17 | pink | yes | SL36 | 45.78 | 15.7 | Zagoršek Tjaša | 132 | 138 | 170 | 172 | 178 | 184 | 281 | 287 | 94 | 94 | 151 | 153 | 147 | 170 | 244 | 246 | 0 | 0 |
| Slovenia | Hap_17 | pink | yes | SL37 | 46.05 | 14.71 | Zagoršek Tjaša | 132 | 134 | 151 | 162 | 178 | 187 | 281 | 281 | 94 | 94 | 172 | 172 | 147 | 170 | 239 | 239 | 276 | 288 |
| Slovenia | Hap_17 | pink | yes | SL38 | 45.77 | 14.69 | Zagoršek Tjaša | 136 | 138 | 170 | 170 | 180 | 183 | 0 | 0 | 94 | 100 | 153 | 159 | 147 | 149 | 239 | 248 | 272 | 276 |
| Slovenia | Hap_17 | pink | yes | SL39 | 45.65 | 14.75 | Zagoršek Tjaša | 125 | 132 | 170 | 172 | 180 | 189 | 0 | 0 | 94 | 94 | 153 | 153 | 170 | 170 | 0 | 0 | 0 | 0 |
| Slovenia | Hap_42 | pink | yes | SL4 | 45.94 | 15.73 | Kryštufek Boris | 129 | 129 | 159 | 162 | 187 | 187 | 281 | 281 | 94 | 94 | 155 | 155 | 147 | 147 | 239 | 246 | 276 | 288 |
| Slovenia | Hap_17 | pink | yes | SL40 | 45.54 | 13.77 | Kryštufek Boris | 129 | 136 | 159 | 170 | 180 | 183 | 285 | 285 | 94 | 100 | 159 | 159 | 147 | 172 | 248 | 248 | 276 | 276 |
| Slovenia | Hap_42 | pink | yes | SL41 | 46.05 | 14.11 | Kryštufek Boris | 129 | 129 | 162 | 172 | 180 | 180 | 281 | 281 | 94 | 100 | 153 | 153 | 147 | 147 | 246 | 246 | 0 | 0 |
| Slovenia | Hap_39 | pink | yes | SL42 | 45.55 | 13.77 | Štáhlavský František, Plíšková Jana | 136 | 138 | 159 | 161 | 184 | 184 | 281 | 281 | 94 | 100 | 153 | 159 | 170 | 170 | 248 | 248 | 279 | 279 |
| Slovenia |  |  | yes | SL43 | 45.54 | 13.77 | Štáhlavský František, Plíšková Jana | 132 | 136 | 159 | 170 | 180 | 183 | 281 | 281 | 94 | 94 | 153 | 157 | 153 | 156 | 246 | 248 | 0 | 0 |
| Slovenia | Hap_17 | pink | yes | SL44 | 45.54 | 13.77 | Hulva Pavel | 129 | 138 | 159 | 170 | 183 | 184 | 281 | 281 | 94 | 94 | 153 | 153 | 168 | 170 | 248 | 248 | 279 | 287 |
| Slovenia | Hap_17 | pink | yes | SL45 | 46.54 | 15.51 | Hulva Pavel | 129 | 129 | 161 | 166 | 184 | 189 | 281 | 281 | 94 | 94 | 153 | 159 | 172 | 178 | 246 | 246 | 278 | 278 |
| Slovenia | Hap_42 | pink | yes | SL5 | 45.56 | 14.21 | Kryštufek Boris | 132 | 136 | 162 | 170 | 180 | 183 | 281 | 281 | 94 | 94 | 151 | 157 | 156 | 172 | 244 | 248 | 276 | 281 |
| Slovenia |  |  | yes | SL7 | 45.56 | 14.21 | Kryštufek Boris | 129 | 129 | 162 | 170 | 180 | 184 | 281 | 285 | 94 | 94 | 153 | 159 | 147 | 172 | 239 | 244 | 276 | 290 |
| Slovenia | Hap_42 | pink | yes | SL8 | 45.34 | 14.52 | Kryštufek Boris | 127 | 145 | 162 | 164 | 176 | 183 | 281 | 281 | 94 | 94 | 153 | 157 | 168 | 170 | 246 | 250 | 276 | 283 |
| Serbia | Hap_20 | green | yes | SR1 | 46.15 | 14.23 | Kryštufek Boris | 138 | 140 | 140 | 159 | 183 | 183 | 281 | 285 | 94 | 94 | 153 | 155 | 144 | 151 | 246 | 248 | 272 | 274 |
| Serbia | Hap_20 | green | yes | SR2 | 46.08 | 14.52 | Kryštufek Boris | 129 | 134 | 155 | 172 | 184 | 187 | 281 | 294 | 94 | 94 | 155 | 157 | 144 | 144 | 246 | 248 | 272 | 272 |
| Serbia | Hap_41 | pink | yes | SR3 | 46.08 | 14.52 | Kryštufek Boris | 123 | 140 | 159 | 166 | 178 | 180 | 281 | 283 | 100 | 100 | 148 | 157 | 156 | 161 | 246 | 246 | 276 | 278 |
| Serbia | Hap_18 | pink | yes | SR4 | 45.87 | 14.82 | Kryštufek Boris | 123 | 134 | 151 | 159 | 187 | 189 | 285 | 285 | 94 | 104 | 0 | 0 | 0 | 0 | 239 | 248 | 0 | 0 |
| Serbia | Hap_17 | pink | yes | SR5 | 46.27 | 14.32 | Kryštufek Boris | 123 | 123 | 159 | 162 | 176 | 178 | 281 | 285 | 94 | 104 | 151 | 157 | 147 | 151 | 246 | 248 | 276 | 279 |
| Serbia |  |  | yes | SR6 | 46.58 | 15.83 | Kryštufek Boris | 117 | 140 | 159 | 166 | 176 | 178 | 281 | 285 | 94 | 104 | 157 | 157 | 149 | 153 | 246 | 248 | 276 | 278 |
| Serbia |  |  | yes | SR7 | 42.38 | 21.98 | Kryštufek Boris | 125 | 129 | 159 | 166 | 178 | 178 | 281 | 281 | 94 | 104 | 155 | 172 | 153 | 153 | 246 | 248 | 274 | 274 |
| Serbia | Hap_46 | pink | yes | SR8 | 42.61 | 21.87 | Kryštufek Boris | 125 | 129 | 159 | 166 | 178 | 178 | 281 | 281 | 94 | 104 | 155 | 172 | 153 | 153 | 246 | 248 | 274 | 274 |
| Vojvodina | Hap_17 | pink | yes | VOJ1 | 44.28 | 22.10 | Kryštufek Boris | 134 | 134 | 159 | 159 | 158 | 176 | 283 | 287 | 94 | 94 | 157 | 157 | 147 | 176 | 250 | 250 | 272 | 279 |
| Vojvodina | Hap_40 | pink | yes | VOJ2 | 44.34 | 22.15 | Kryštufek Boris | 134 | 134 | 159 | 159 | 183 | 189 | 285 | 287 | 94 | 94 | 151 | 157 | 147 | 166 | 248 | 248 | 270 | 274 |
| Czech republic | Hap_27 | orange | yes | 1 | 44.40 | 22.21 | Bolfíková Barbora, Hulva Pavel, Kaftan Milan | 119 | 132 | 162 | 164 | 176 | 180 | 281 | 281 | 94 | 94 | 151 | 159 | 144 | 144 | 246 | 248 | 272 | 281 |
| Czech republic | Hap_29 | orange | yes | 5 | 44.40 | 22.22 | Bolfíková Barbora, Hulva Pavel, Kaftan Milan | 119 | 134 | 162 | 164 | 180 | 180 | 281 | 289 | 94 | 94 | 144 | 157 | 144 | 153 | 250 | 250 | 272 | 278 |
| Czech republic | |  | yes | 7 | 44.40 | 22.22 | Bolfíková Barbora, Hulva Pavel | 132 | 138 | 164 | 164 | 179 | 184 | 289 | 289 | 94 | 94 | 148 | 157 | 144 | 144 | 248 | 250 | 272 | 278 |
| Czech republic | Hap_27 | orange | yes | 8 | 44.42 | 22.25 | Lučan Radek | 132 | 132 | 161 | 161 | 179 | 191 | 281 | 289 | 94 | 94 | 157 | 159 | 144 | 144 | 250 | 250 | 278 | 281 |
| Czech republic | Hap_27 | orange | yes | 9 | 44.90 | 21.43 | Lučan Radek | 132 | 132 | 159 | 166 | 174 | 176 | 281 | 281 | 94 | 94 | 148 | 148 | 144 | 176 | 248 | 250 | 272 | 270 |
| Czech republic | Hap_27 | orange | yes | 10 | 44.75 | 20.98 | Lučan Radek | 119 | 132 | 162 | 164 | 0 | 0 | 281 | 289 | 94 | 94 | 144 | 148 | 144 | 170 | 248 | 250 | 272 | 278 |
| Czech republic | Hap_27 | orange | yes | 14 | 50.03 | 14.43 | Bolfíková Barbora, Hulva Pavel | 129 | 129 | 162 | 164 | 183 | 191 | 281 | 281 | 94 | 94 | 157 | 159 | 168 | 170 | 248 | 250 | 276 | 283 |
| Czech republic | Hap_27 | orange | yes | 16 | 49.98 | 15.25 | Bolfíková Barbora | 132 | 138 | 159 | 164 | 176 | 176 | 281 | 289 | 94 | 94 | 159 | 159 | 144 | 144 | 248 | 248 | 272 | 272 |
| Czech republic | Hap_27 | orange | yes | 17 | 50.08 | 14.35 | Gvoždík Václav | 119 | 119 | 164 | 164 | 174 | 180 | 289 | 289 | 94 | 94 | 151 | 153 | 144 | 144 | 246 | 248 | 272 | 281 |
| Czech republic | |  | yes | 18 | 50.43 | 14.32 | Gvoždík Václav | 136 | 136 | 166 | 166 | 180 | 180 | 281 | 289 | 94 | 94 | 144 | 157 | 144 | 176 | 246 | 250 | 278 | 283 |
| Czech republic | Hap_27 | orange | yes | 21 | 50.43 | 14.32 | Bolfíková Barbora, Hulva Pavel | 132 | 138 | 159 | 164 | 176 | 180 | 283 | 289 | 94 | 94 | 159 | 159 | 144 | 144 | 246 | 248 | 270 | 278 |
| Czech republic | Hap_27 | orange | yes | 22 | 50.28 | 14.8 | Bolfíková Barbora, Hulva Pavel | 132 | 138 | 159 | 164 | 184 | 189 | 281 | 290 | 94 | 94 | 153 | 153 | 144 | 144 | 248 | 248 | 270 | 281 |
| Czech republic | Hap_27 | orange | yes | 24 | 48.78 | 16.78 | Bolfíková Barbora, Schneiderová Irena | 132 | 134 | 159 | 161 | 180 | 185 | 283 | 289 | 94 | 94 | 144 | 157 | 144 | 144 | 246 | 250 | 274 | 279 |
| Czech republic | Hap_27 | orange | yes | 25 | 50.08 | 14.35 | Bolfíková Barbora, Schneiderová Irena | 132 | 134 | 164 | 168 | 176 | 285 | 289 | 289 | 94 | 94 | 144 | 144 | 144 | 144 | 248 | 250 | 270 | 276 |
| Czech republic | Hap_27 | orange | yes | 26 | 50.10 | 14.42 | Bolfíková Barbora, Schneiderová Irena | 132 | 132 | 159 | 162 | 180 | 180 | 283 | 289 | 94 | 94 | 144 | 157 | 144 | 144 | 246 | 248 | 278 | 283 |
| Czech republic | Hap_27 | orange | yes | 27 | 50.40 | 14.43 | Bolfíková Barbora, Schneiderová Irena | 119 | 132 | 159 | 168 | 176 | 184 | 289 | 289 | 94 | 94 | 144 | 151 | 144 | 174 | 248 | 250 | 270 | 270 |
| Czech republic | Hap_27 | orange | yes | 28 | 50.08 | 14.35 | Bolfíková Barbora, Schneiderová Irena | 132 | 132 | 159 | 161 | 180 | 184 | 281 | 289 | 94 | 94 | 144 | 151 | 144 | 144 | 246 | 246 | 272 | 281 |
| Czech republic | Hap_27 | orange | yes | 29 | 50.08 | 14.35 | Bolfíková Barbora, Schneiderová Irena | 119 | 134 | 166 | 166 | 184 | 184 | 290 | 290 | 94 | 94 | 144 | 159 | 144 | 174 | 246 | 250 | 274 | 274 |
| Czech republic | Hap_27 | orange | yes | 32 | 50.02 | 14.48 | Šobotník Jan | 132 | 132 | 161 | 166 | 184 | 184 | 281 | 281 | 94 | 94 | 144 | 144 | 147 | 170 | 246 | 248 | 270 | 276 |
| Czech republic | Hap_41 | orange | yes | 43 | 50.02 | 14.48 | Gvoždík Václav | 132 | 132 | 161 | 169 | 180 | 180 | 281 | 281 | 94 | 94 | 144 | 144 | 142 | 144 | 248 | 248 | 278 | 279 |
| Czech republic | |  | yes | 44 | 50.02 | 14.48 | Gvoždík Václav | 119 | 132 | 161 | 164 | 180 | 183 | 281 | 281 | 94 | 94 | 144 | 157 | 144 | 144 | 248 | 248 | 272 | 279 |
| Czech republic | Hap_27 | orange | yes | 47 | 50.02 | 14.48 | Bolfíková Barbora | 119 | 119 | 164 | 166 | 180 | 197 | 281 | 285 | 94 | 94 | 144 | 153 | 144 | 144 | 248 | 248 | 281 | 283 |
| Czech republic | Hap_27 | orange | yes | 58 | 50.02 | 14.48 | Gvoždík Václav | 132 | 134 | 159 | 161 | 191 | 193 | 289 | 290 | 94 | 94 | 151 | 153 | 144 | 176 | 248 | 248 | 283 | 283 |
| Czech republic | Hap_27 | orange | yes | 59 | 50.02 | 14.48 | Gvoždík Václav | 132 | 134 | 159 | 161 | 180 | 193 | 289 | 290 | 94 | 94 | 144 | 153 | 144 | 144 | 246 | 248 | 283 | 283 |
| Czech republic | Hap_27 | orange | yes | 84 | 49.35 | 18.27 | Dvorská Zdena | 138 | 138 | 159 | 159 | 180 | 183 | 283 | 289 | 94 | 94 | 159 | 159 | 144 | 170 | 248 | 248 | 278 | 281 |
| Czech republic | Hap_27 | orange | yes | 85 | 50.10 | 14.60 | Dvorská Zdena | 132 | 132 | 164 | 164 | 180 | 183 | 285 | 285 | 94 | 94 | 144 | 159 | 144 | 144 | 248 | 250 | 279 | 281 |
| Czech republic | Hap_42 | orange | yes | 96 | 50.10 | 14.60 | Reiter Antonín | 132 | 138 | 164 | 166 | 172 | 183 | 283 | 283 | 94 | 94 | 144 | 151 | 144 | 144 | 246 | 246 | 270 | 276 |
| Czech republic | Hap_41 | orange | yes | 98 | 50.12 | 14.42 | Reiter Antonín | 132 | 132 | 159 | 161 | 176 | 180 | 281 | 283 | 94 | 94 | 157 | 157 | 144 | 153 | 246 | 246 | 276 | 278 |
| Czech republic | Hap_27 | orange | yes | 101 | 50.12 | 14.48 | Hulva Pavel | 132 | 132 | 159 | 161 | 176 | 180 | 281 | 283 | 94 | 94 | 151 | 151 | 144 | 144 | 241 | 246 | 270 | 270 |
| Czech republic | |  | yes | 111 | 50.12 | 14.48 | Pithartová Tereza | 132 | 132 | 161 | 166 | 180 | 180 | 285 | 285 | 94 | 94 | 153 | 157 | 144 | 144 | 246 | 246 | 272 | 274 |
| Czech republic | Hap_27 | orange | yes | 112 | 50.12 | 14.48 | Pithartová Tereza | 119 | 132 | 164 | 164 | 180 | 184 | 281 | 289 | 94 | 94 | 151 | 157 | 144 | 174 | 246 | 246 | 272 | 281 |
| Czech republic | Hap_27 | orange | yes | 113 | 50.03 | 14.43 | Pithartová Tereza | 119 | 138 | 175 | 175 | 176 | 197 | 281 | 289 | 94 | 94 | 151 | 157 | 144 | 174 | 246 | 248 | 270 | 281 |
| Czech republic | Hap_27 | orange | yes | 116 | 48.85 | 16.3 | Pithartová Tereza | 132 | 132 | 159 | 159 | 180 | 197 | 281 | 283 | 94 | 94 | 144 | 144 | 144 | 174 | 246 | 248 | 270 | 283 |
| Czech republic | Hap_27 | orange | yes | 117 | 48.90 | 16.20 | Pithartová Tereza | 119 | 119 | 161 | 166 | 176 | 180 | 281 | 289 | 94 | 94 | 144 | 153 | 144 | 144 | 246 | 248 | 270 | 272 |
| Czech republic | Hap_27 | orange | yes | 121 | 50.15 | 14.73 | Pithartová Tereza | 132 | 132 | 161 | 164 | 184 | 197 | 285 | 289 | 94 | 94 | 144 | 157 | 144 | 168 | 246 | 250 | 281 | 283 |
| Czech republic | Hap_27 | orange | yes | 123 | 50.12 | 14.42 | Kaftan Milan | 132 | 132 | 166 | 166 | 180 | 184 | 289 | 289 | 94 | 94 | 144 | 144 | 144 | 174 | 246 | 250 | 272 | 281 |
| Czech republic | Hap_27 | orange | yes | 124 | 50.12 | 14.42 | Kaftan Milan | 132 | 132 | 162 | 164 | 184 | 184 | 289 | 289 | 94 | 94 | 144 | 144 | 144 | 144 | 248 | 248 | 270 | 274 |
| Czech republic | Hap_27 | orange | yes | 129 | 50.12 | 14.42 | Dvorská Zdena | 132 | 132 | 161 | 161 | 176 | 184 | 281 | 290 | 94 | 94 | 151 | 151 | 144 | 144 | 250 | 250 | 270 | 274 |
| Czech republic | Hap_27 | orange | yes | 135 | 50.12 | 14.42 | Zoo Olomouc | 132 | 140 | 159 | 166 | 176 | 176 | 281 | 299 | 94 | 94 | 144 | 144 | 147 | 147 | 246 | 248 | 281 | 281 |
| Czech republic | Hap_28 | orange | yes | 139 | 50.12 | 14.42 | Baláž Vojto | 129 | 132 | 159 | 166 | 176 | 176 | 0 | 0 | 94 | 94 | 144 | 151 | 144 | 172 | 250 | 250 | 0 | 0 |
| Czech republic | |  | yes | 142 | 50.15 | 14.73 | Baláž Vojto | 132 | 132 | 161 | 161 | 180 | 180 | 281 | 281 | 94 | 94 | 144 | 144 | 144 | 144 | 248 | 250 | 270 | 281 |
| Czech republic | Hap_27 | orange | yes | 144 | 50.07 | 14.42 | Gvoždík Václav | 132 | 132 | 161 | 164 | 191 | 193 | 281 | 290 | 94 | 94 | 144 | 153 | 176 | 176 | 248 | 250 | 270 | 283 |
| Czech republic | Hap_27 | orange | yes | 147 | 50.07 | 14.42 | ZS Pavlov | 132 | 138 | 159 | 164 | 162 | 176 | 281 | 281 | 94 | 94 | 159 | 159 | 144 | 168 | 246 | 250 | 274 | 278 |
| Czech republic | Hap_27 | orange | yes | 149 | 50.53 | 14.12 | ZS Pavlov | 132 | 132 | 164 | 164 | 176 | 184 | 283 | 289 | 94 | 94 | 151 | 153 | 135 | 138 | 246 | 250 | 278 | 283 |
| Czech republic | Hap_27 | orange | yes | 164 | 49.58 | 17.25 | Falco Dolní Týnec | 132 | 132 | 159 | 164 | 180 | 189 | 289 | 290 | 94 | 94 | 151 | 151 | 144 | 144 | 246 | 248 | 281 | 281 |
| Czech republic | Hap_27 | orange | yes | 168 | 49.07 | 17.43 | Falco Dolní Týnec | 132 | 132 | 159 | 161 | 180 | 184 | 281 | 290 | 94 | 94 | 144 | 144 | 144 | 172 | 246 | 250 | 279 | 281 |
| Czech republic | Hap_27 | orange | yes | 183 | 50.12 | 14.42 | Zoo Chomutov | 132 | 132 | 159 | 159 | 189 | 191 | 281 | 281 | 94 | 94 | 151 | 151 | 144 | 170 | 248 | 250 | 270 | 279 |
| Czech republic | Hap_27 | orange | yes | 209 | 50.12 | 14.48 | Bolfíková Barbora, Hulva Pavel, Janko Karel | 132 | 134 | 159 | 159 | 183 | 183 | 281 | 297 | 94 | 94 | 144 | 157 | 151 | 170 | 248 | 248 | 270 | 270 |
| Czech republic | Hap_27 | orange | yes | 214 | 49.35 | 15.93 | ZS Bartošovice | 132 | 132 | 164 | 166 | 180 | 183 | 281 | 281 | 94 | 94 | 144 | 157 | 144 | 174 | 248 | 248 | 270 | 274 |
| Slovakia | Hap_41 | orange | yes | sk7 | 49.67 | 15.40 | Ferenc Michal | 119 | 134 | 161 | 161 | 0 | 0 | 281 | 281 | 94 | 94 | 144 | 157 | 138 | 172 | 248 | 250 | 276 | 278 |
| Slovakia | Hap_1 | yellow | yes | sk8 | 50.53 | 14.12 | Ferenc Michal | 134 | 136 | 159 | 165 | 183 | 184 | 285 | 290 | 94 | 94 | 153 | 157 | 138 | 178 | 248 | 248 | 274 | 276 |
| Slovakia | Hap_1 | yellow | yes | sk9 | 50.53 | 14.12 | Ferenc Michal | 119 | 132 | 159 | 166 | 183 | 184 | 281 | 281 | 94 | 94 | 148 | 157 | 153 | 153 | 246 | 246 | 270 | 278 |
| Slovakia |  |  | yes | sk11 | 50.45 | 13.40 | Ferenc Michal | 134 | 140 | 161 | 161 | 183 | 184 | 291 | 290 | 94 | 94 | 144 | 144 | 178 | 180 | 246 | 248 | 278 | 278 |
| Slovakia | Hap_27 | orange | yes | sk12 | 49.85 | 18.37 | Varadinová Zuzana | 132 | 148 | 159 | 159 | 189 | 199 | 281 | 285 | 94 | 94 | 151 | 161 | 149 | 153 | 246 | 248 | 270 | 278 |
| Slovakia | Hap_1 | yellow | yes | sk13 | 49.77 | 18.30 | Mikulíček Petr | 129 | 129 | 162 | 166 | 183 | 184 | 281 | 290 | 94 | 94 | 151 | 151 | 168 | 174 | 246 | 248 | 272 | 276 |
| Slovakia | Hap_27 | orange | yes | sk14 | 48.43 | 19.65 | Mikulíček Petr | 129 | 134 | 161 | 166 | 184 | 184 | 283 | 289 | 94 | 96 | 148 | 161 | 153 | 168 | 248 | 250 | 274 | 274 |
| Slovakia | Hap_27 | orange | yes | sk15 | 48.42 | 19.78 | Mikulíček Petr | 129 | 132 | 159 | 162 | 179 | 183 | 281 | 281 | 94 | 94 | 148 | 155 | 170 | 172 | 246 | 248 | 274 | 281 |
| Slovakia | Hap_27 | orange | yes | sk16 | 48.22 | 19.82 | Celuch Martin | 132 | 132 | 159 | 165 | 176 | 184 | 281 | 281 | 94 | 94 | 148 | 151 | 170 | 172 | 248 | 250 | 276 | 281 |
| Slovakia | Hap_27 | orange | yes | sk17 | 48.22 | 19.82 | Celuch Martin | 132 | 134 | 162 | 162 | 184 | 191 | 281 | 281 | 94 | 94 | 157 | 157 | 144 | 144 | 246 | 248 | 274 | 283 |
| Slovakia | Hap_1 | yellow | yes | sk20 | 48.15 | 17.10 | Celuch Martin | 136 | 138 | 162 | 164 | 181 | 181 | 289 | 289 | 94 | 94 | 144 | 155 | 144 | 144 | 246 | 248 | 272 | 278 |
| Slovakia | Hap_27 | yellow | yes | sk21 | 48.73 | 19.37 | Celuch Martin | 136 | 136 | 162 | 166 | 176 | 183 | 281 | 289 | 94 | 94 | 146 | 155 | 144 | 144 | 246 | 248 | 276 | 279 |
| Slovakia | Hap_1 | yellow | yes | sk22 | 48.72 | 19.13 | Celuch Martin | 121 | 136 | 159 | 166 | 170 | 183 | 283 | 285 | 94 | 94 | 153 | 159 | 138 | 144 | 246 | 248 | 283 | 283 |
| Slovakia | Hap_2 | yellow | yes | sk23 | 48.67 | 17.35 | Celuch Martin | 136 | 138 | 164 | 165 | 184 | 187 | 281 | 285 | 94 | 94 | 153 | 159 | 151 | 153 | 246 | 248 | 278 | 283 |
| Slovakia | Hap_3 | yellow | yes | sk24 | 48.15 | 17.10 | Celuch Martin | 119 | 132 | 166 | 166 | 176 | 176 | 281 | 294 | 94 | 94 | 144 | 163 | 151 | 174 | 246 | 250 | 272 | 278 |
| Slovakia | Hap_2 | yellow | yes | sk25 | 48.67 | 17.25 | Benda Petr | 134 | 136 | 159 | 159 | 181 | 184 | 281 | 290 | 94 | 94 | 144 | 144 | 144 | 153 | 246 | 246 | 276 | 278 |
| Slovakia | Hap_8 | yellow | yes | sk28 | 49.31 | 21.21 | Benda Petr | 132 | 134 | 159 | 161 | 183 | 183 | 281 | 290 | 94 | 94 | 144 | 144 | 138 | 174 | 246 | 246 | 278 | 278 |
| Slovakia | Hap_1 | yellow | yes | sk29 | 48.99 | 21.24 | Bolfíková Barbora | 132 | 138 | 159 | 166 | 183 | 183 | 290 | 290 | 94 | 94 | 153 | 159 | 144 | 172 | 250 | 250 | 274 | 278 |
| Slovakia |  |  | yes | sk30 | 49.09 | 21.31 | Bolfíková Barbora | 132 | 138 | 162 | 162 | 183 | 187 | 283 | 298 | 94 | 94 | 144 | 157 | 153 | 170 | 248 | 248 | 274 | 276 |
| Slovakia | Hap_27 | orange | yes | sk34 | 49.09 | 21.31 | Noga Michal | 134 | 134 | 159 | 159 | 174 | 183 | 281 | 281 | 94 | 94 | 151 | 151 | 138 | 170 | 248 | 250 | 274 | 276 |
| Slovakia | Hap_42 | orange | yes | sk44 | 48.75 | 21.27 | Bolfíková Barbora | 129 | 132 | 159 | 159 | 183 | 184 | 278 | 281 | 94 | 94 | 148 | 151 | 168 | 170 | 250 | 250 | 274 | 276 |
| Slovakia | Hap_1 | yellow | yes | sk48 | 48.96 | 20.51 | Hájková Andrea | 127 | 127 | 159 | 166 | 187 | 187 | 281 | 281 | 94 | 94 | 144 | 157 | 144 | 153 | 248 | 248 | 272 | 281 |
| Slovakia | Hap_1 | yellow | yes | sk49 | 49.09 | 21.31 | Hájková Andrea | 127 | 134 | 159 | 166 | 183 | 184 | 281 | 281 | 94 | 94 | 144 | 157 | 144 | 144 | 246 | 246 | 274 | 276 |
| Slovakia | Hap_1 | yellow | yes | sk50 | 48.75 | 19.66 | Hájková Andrea | 127 | 127 | 159 | 166 | 183 | 184 | 281 | 281 | 94 | 94 | 155 | 157 | 144 | 172 | 248 | 248 | 274 | 276 |
| Slovakia | Hap_1 | yellow | yes | sk51 | 48.18 | 18.36 | Hájková Andrea | 127 | 134 | 159 | 166 | 183 | 184 | 281 | 281 | 94 | 94 | 155 | 157 | 153 | 172 | 246 | 248 | 274 | 276 |
|  |  |  |  |  |  |  |  |  |  |  |  |  |  |  |  |  |  |  |  |  |  |  |  |  |  |

Table of all used samples. Given are: country of origin, haplotype code, colour code in Geneland analysis, sucess of microsatellite gonotyping, used code for individual, coordinates, collectors and allele scores of microsatellites.
