# Supplementary material for: Glacial allopatry vs. postglacial parapatry and peripatry: the case of hedgehogs
Source: PeerJ. 2017 Apr 25;5:e3163. doi: 10.7717/peerj.3163 (PMC5407276; doi:10.7717/peerj.3163)
Supplement: Table S2 — Number of alleles (Na), allelic richness ( AR), expected heterozygosity ( HE), observed heterozygosity ( HO), inbreeding coefficient ( FIS) are given for each population. [file peerj-05-3163-s002.docx]

|  | *N*_a_ | *A*_R_ | *H*_E_ | *H*_O_ | *F*_IS_ |
| --- | --- | --- | --- | --- | --- |
| Crete | 6 | 4.493 | 0.5243 | 0.4292 | 0.170 |
| Czech republic | 7 | 5.943 | 0.6329 | 0.5468 | 0.134 |
| Balkan, Romania, Slovakia, Hungary, Slovenia | 15 | 8.222 | 0.7609 | 0.6415 | 0.164 |
|  |  |  |  |  |  |
| The descriptive statistics of microsatellite genetic diversity in three populations of *Erinaceus roumanicus*, which were recognised by the Bayesian analysis using Structure. Number of alleles (*N*_a_), allelic richness (*A*_R_), expected heterozygosity (*H*_E_), observed heterozygosity (*H*_O_), inbreeding coefficient (*F*_IS_) are given for each population. | | | | | |
