# Supplementary material for: Glacial allopatry vs. postglacial parapatry and peripatry: the case of hedgehogs
Source: PeerJ. 2017 Apr 25;5:e3163. doi: 10.7717/peerj.3163 (PMC5407276; doi:10.7717/peerj.3163)
Supplement: Table S3 — Table of microsatellite loci (Locus), their accession numbers (GenBank accession no.), annealing temperatures (Annealing temp.), fluorescent labels and repeat motifs. For control region, primers and annealing temperature are given. [file peerj-05-3163-s003.docx]

| **Microsatellite loci** | |  |  |  |
| --- | --- | --- | --- | --- |
| Locus | GenBank accession no. | Annealing temp. (°C) | fluorescent label | Repeat motif |
| EEU2 | U63911 | 62 | PET | d(CA)18 |
| EEU3 | U63912 | 62 | FAM | d(CA)18 |
| EEU4 | U63913 | 62 | NED | d(CA)19 |
| EEU5 | U63914 | 56 | VIC | d(CA)24 |
| EEU6 | U63915 | 56 | PET | d(GA)18 |
| EEU12H | AF276820 | 64 | FAM | d(CT)15 |
| EEU37H | AF276823 | 64 | VIC | d(GT)23 |
| EEU43H | AF276824 | 56 | FAM | d(GT)25 (GA)6 |
| EEU54H | AF276825 | 56 | NED | d(GA)31 |
|  |  |  |  |  |
| **Control region** | |  |  |  |
| primer | sequence |  | Annealing temp. (°C) | |
| ProL-He | 5′-ATACTCCTACCATCAACACCCAAAG-3′ | | 60 |  |
| DLH-He | 5′-GTCCTGAAGAAAGAACCAGATGTC-3′ | |  |  |
